# The MX-Helix of Muscle nAChR Subunits Regulates Receptor Assembly and Surface Trafficking

**DOI:** 10.3389/fnmol.2020.00048

**Published:** 2020-03-24

**Authors:** Jolene Chang Rudell, Lucia Soares Borges, Vladimir Yarov-Yarovoy, Michael Ferns

**Affiliations:** ^1^Department of Physiology and Membrane Biology, University of California, Davis, Davis, CA, United States; ^2^Department of Anesthesiology and Pain Medicine, University of California, Davis, Davis, CA, United States

**Keywords:** nicotinic acetylcholine receptor, protein motif, trafficking, neuromuscular junction, congenital myasthenic syndrome

## Abstract

Nicotinic acetylcholine receptors (AChRs) are pentameric channels that mediate fast transmission at the neuromuscular junction (NMJ) and defects in receptor expression underlie neuromuscular disorders such as myasthenia gravis and congenital myasthenic syndrome (CMS). Nicotinic receptor expression at the NMJ is tightly regulated and we previously identified novel Golgi-retention signals in the β and δ subunit cytoplasmic loops that regulate trafficking of the receptor to the cell surface. Here, we show that the Golgi retention motifs are localized in the MX-helix, a juxta-membrane alpha-helix present in the proximal cytoplasmic loop of receptor subunits, which was defined in recent crystal structures of cys-loop receptor family members. First, mutational analysis of CD4-MX-helix chimeric proteins showed that the Golgi retention signal was dependent on both the amphipathic nature of the MX-helix and on specific lysine residues (βK353 and δK351). Moreover, retention was associated with ubiquitination of the lysines, and βK353R and δK351R mutations reduced ubiquitination and increased surface expression of CD4-β and δ MX-helix chimeric proteins. Second, mutation of these lysines in intact β and δ subunits perturbed Golgi-based glycosylation and surface trafficking of the AChR. Notably, combined βK353R and δK351R mutations increased the amount of surface AChR with immature forms of glycosylation, consistent with decreased Golgi retention and processing. Third, we found that previously identified CMS mutations in the ε subunit MX-helix decreased receptor assembly and surface levels, as did an analogous mutation introduced into the β subunit MX-helix. Together, these findings indicate that the subunit MX-helix contributes to receptor assembly and is required for normal expression of the AChR and function of the NMJ. In addition, specific determinants in the β and δ subunit MX-helix contribute to quality control of AChR expression by intracellular retention and ubiquitination of unassembled subunits, and by facilitating the appropriate glycosylation of assembled surface AChR.

## Introduction

The muscle nicotinic acetylcholine receptor (AChR) is a ligand-gated ion channel belonging to the cys-loop superfamily of receptors that includes the neuronal ACh, GABA_*A*_, glycine and 5HT_3_ receptors. The muscle AChR mediates fast transmission at the neuromuscular junction (NMJ) and is a pentamer composed of four subunits with the stoichiometry α_2_βεδ (adult form). All of the receptor subunits are homologous and have a large extracellular N-terminal domain, 4 transmembrane domains and a large cytoplasmic loop between transmembrane domains 3 and 4 (Unwin, 2005; Sine, 2012). High resolution structures have recently been obtained for several members of the cys-loop family and reveal a conserved architecture of the channel and constituent subunits, as well as providing new insights into the structural basis of transmitter binding and channel gating (Unwin, 2005; Hassaine et al., 2014; Miller and Aricescu, 2014; Huang et al., 2015; Wu et al., 2015; Morales-Perez et al., 2016). One region that is only partially resolved in these structures, however, is the subunit large cytoplasmic loop domain, which contains critical determinants for trafficking and targeting of the receptor (Millar and Harkness, 2008; Albuquerque et al., 2009). This region begins with the short MX helix located just after TM3 and ends with the long MA helix that is continuous with TM4, but no structure is available for the intervening sequence (Wu et al., 2015).

Synaptic transmission at the NMJ is rapid and reliable due in part to the high density of AChRs in the postsynaptic membrane, and defects in AChR expression in genetic and autoimmune diseases impair transmission and result in myasthenia (Verschuuren et al., 2016; Engel, 2018). The expression of AChR at the NMJ is tightly regulated and depends on the appropriate assembly, trafficking and localization of receptors in the postsynaptic membrane (Millar and Harkness, 2008). This slow and complex process begins with the synthesis and assembly of receptor subunits in the ER. Assembled receptor is then exported from the endoplasmic reticulum (ER) and trafficked to the Golgi apparatus for further processing, which includes modification of N-linked oligosaccharides on the γ and δ subunit to more complex forms (Gu et al., 1989; St John, 2009). Finally, the mature AChR is sorted, trafficked to the plasma membrane, and localized in the postsynaptic membrane. Each step in this process is thought to be directed by specific molecular signals in the receptor subunits that function in distinct cellular compartments. Moreover, as the process is relatively inefficient with only 10–30% of the synthesized subunits being incorporated into surface nAChR (Merlie and Lindstrom, 1983; Wanamaker et al., 2003), quality control mechanisms play an important role in ensuring that only correctly assembled, functional channels are expressed on the cell surface (Fu et al., 2016).

The molecular basis for the initial steps in AChR biogenesis are relatively well understood, with several signals being defined on the receptor subunits that govern receptor assembly and ER quality control (Millar and Harkness, 2008; Colombo et al., 2013). For example, determinants in the subunit extracellular domains direct the ordered assembly of receptor subunits, helping ensure the correct stoichiometry of receptor. Other determinants in the subunit first transmembrane domain and major cytoplasmic loop permit the export of pentameric receptor from the ER, but retain unassembled or partially assembled subunits (Keller et al., 2001; Wang et al., 2002; Ren et al., 2005), where they are then ubiquitinated and degraded by the ER-associated degradation machinery (Christianson and Green, 2004). In contrast, little is known about the molecular signals that regulate subsequent steps in AChR trafficking. Golgi trafficking is likely regulated, as a genetic screen in *Caenorhabditis elegans* identified the Golgi-resident protein, unc-50, as being required for trafficking of one subtype of nAChR to the NMJ (Eimer et al., 2007). Moreover, it likely provides an additional quality control checkpoint, as mutations in the α subunit loop that allow ER export result in the unassembled subunit being retained in the Golgi by an unknown mechanism (Keller et al., 2001). To define the molecular determinants that regulate these later trafficking steps we performed an unbiased screen for post-ER trafficking signals in the AChR subunit major cytoplasmic loop. We identified novel motifs in the β and δ subunit loops that mediate Golgi retention, and mutation of this motif permits surface expression of unassembled subunit loops and increases surface levels of assembled AChR (Rudell et al., 2014). Here, we show that the Golgi retention signal is localized in the MX-alpha helix and is centered on key lysine residues. We find that the motif contributes to quality control both through ubiquitination and intracellular retention of unassembled subunits, and by facilitating the appropriate Golgi-based glycosylation of assembled receptor. In addition, we identify distinct determinants in the MX helix that contribute to receptor assembly, and CMS-linked mutations in this region impair subunit assembly and AChR expression. Thus, the MX-helix of receptor subunits contain important molecular signals that regulate the assembly, trafficking and expression of muscle AChR at the NMJ.

## Materials and Methods

### Rosetta Molecular Modeling

Homology modeling of human AChR beta and delta subunits was performed using Rosetta structural modeling software (Rohl et al., 2004; Song et al., 2013; Bender et al., 2016; Alford et al., 2017) based Robetta server (Park et al., 2018) and x-ray structure of the human alpha4beta2 nicotinic receptor (Morales-Perez et al., 2016) as a template. Human AChR beta subunit was modeled based on human alpha4beta2 nicotinic receptor beta 2 subunit (PDB ID: 5KXI chain C) (Morales-Perez et al., 2016). Human AChR delta subunit was modeled based on human alpha4beta2 nicotinic receptor beta 2 subunit (PDB ID: 5KXI chain B) (Morales-Perez et al., 2016). 1,000 models were generated for each subunit and clustered (Bonneau et al., 2002) to identify top 5 models. We then superimposed a representative model from one of the top human AChR beta subunit models onto human alpha4beta2 nicotinic receptor beta 2 subunit (PDB ID: 5KXI chain C) and a representative model from one of the top human AChR delta subunit models onto human alpha4beta2 nicotinic receptor beta 2 subunit (PDB ID: 5KXI chain B) in the complete structure of the human alpha4beta2 nicotinic receptor (Morales-Perez et al., 2016). All structural figures were generated using the UCSF Chimera package (Pettersen et al., 2004).

### CD4-Subunit Loop Constructs

Chimeric constructs consisting of mouse CD4 extracellular and transmembrane domains fused with the major cytoplasmic loop of each mouse nAChR subunit were generated and described previously (Borges et al., 2008; Rudell et al., 2014). Mutations in the intracellular loops were introduced using the QuikChange Lightning site-directed mutagenesis kit (Agilent Tech.) and confirmed by sequencing.

### Cell Culture and Transfection

HEK cells were maintained in growth media (DMEM supplemented with 10% fetal bovine serum, 2 mM L-glutamine and 200 U/ml penicillin-streptomycin) at 37°C and 5% CO_2_. They were transfected at ∼70% confluence using calcium phosphate (for large 10 cm dishes) or Lipofectamine 3000 (Invitrogen).

C2 mouse muscle cells were maintained in growth media (DMEM supplemented with 20% fetal bovine serum, 0.5% chick embryo extract, 2 mM L-glutamine and 200 U/ml penicillin-streptomycin) at 37°C and 8% CO_2_. Myoblasts were transfected at ∼60–70% confluence using Fugene (Roche) or Lipofectamine 3000 (Invitrogen). Upon reaching confluence the cells were incubated with fusion medium (DMEM supplemented with 5% horse serum and 2 mM L-glutamine) and allowed to differentiate into myotubes for 3–4 days prior to analysis (Borges et al., 2008).

### Surface Expression of CD4 Chimeras and AChR

For on cell western assays, C2 muscle cells were grown on 8-well chamber slides and duplicate wells were transfected with each CD4-subunit loop chimera. After 3–4 days for expression the cells were fixed and one of the two wells was permeabilized with 0.5% Triton X-100. The slides were then incubated with anti-CD4 antibody to assay surface levels of CD4-chimera in non-permeabilized cells (first well), compared to total levels (surface + intracellular) of the same CD4-chimera in permeabilized cells (second well). Bound antibodies were detected using IRDye-conjugated anti-rat secondary antibodies and an Odyssey Imaging System (LiCor). Total signal intensity was measured for each well and the percentage of surface (non-permeabilized) versus total (permeabilized) expression calculated for each CD4-subunit loop chimera. Data was collected from 4–6 independent experiments for each construct.

To assay levels of surface AChR, HEK cells were grown on 6-well plates and transfected with mouse AChR subunits. After 1 d for expression, the cells were labeled with 10 nM I^125^-labeled-α-BuTx (Perkin Elmer) for 45 min. Non-specific binding was determined by treating myotubes with 1 μM of cold α-BuTx for 30 min prior to incubation with I^125^-α-BuTx. Cells were then washed three times with growth media to remove unbound I^125^-α-BuTx, solubilized in 0.1N NaOH and the I^125^-α-BuTx bound to surface AChR was measured with a Packard gamma counter. Background counts were subtracted from the experimental counts and values are reported as a percentage of the total surface counts for cells transfected with wild type AChR.

### Isolation and Immunoblotting of CD4-Chimeras and AChR

To assay ubiquitination of CD4 subunit loop chimeras by western blotting, transfected C2 muscle cells were washed, scraped off and pelleted in ice cold PBS. They were then re-suspended in extraction buffer (0.5% Triton X-100, 25 mM Tris, 25 mM glycine, 150 mM NaCl, 5 mM EDTA, and protease inhibitors) and incubated for 10 min on ice, after which the insoluble proteins were pelleted by centrifugation at 13,000 rpm for 5 min. The CD4 chimeras were immunoprecipitated from the soluble fraction with monoclonal antibody GK1.5 (BD Biosciences-Pharmingen, San Jose, CA) chemically cross-linked to protein G-agarose (Invitrogen, Carlsbad, CA). The isolates were separated on 10% polyacrylamide gels and immunoblotted with anti-ubiquitin antibody P4D1 (Santa Cruz Biotech.). The bound antibodies were then detected using IRDye-conjugated secondary antibody, imaged with an Odyssey Imaging System, and band intensities quantified using ImageStudio (Li-Cor). Immunoblots were re-probed with rmCD4 antibody (R&D Systems) to confirm levels of the different CD4 subunit loop chimeras.

To assay AChR glycosylation, heterologous HEK cells were transfected with wild type or mutant AChR subunits. Epitope tagged (142) delta subunits were used to allow unambiguous identification of delta, and G1G2 delta variants had T78A and S145A mutations to eliminate the two N-linked glycosylation sites (Ramanathan and Hall, 1999). After 1 day for expression, the cells were incubated live with biotinylated α-bungarotoxin (α-BuTx) for 45 min to label surface AChR, and then washed, collected and extracted in buffer containing 0.5% Triton X-100, 25 mM Tris, 25 mM glycine, 150 mM NaCl, 5 mM EDTA and Halt protease inhibitor cocktail (ThermoScientific). First, biotin-α-BuTx-labeled surface receptor was isolated from the extracts using streptavidin-beads (Invitrogen). Then, unlabeled intracellular AChR was isolated from the remaining supernatant by re-incubation with biotin-α-BuTX and pulldown on streptavidin beads. The samples were separated on large format 10% polyacrylamide gels (14 × 14 cm) and immuno-blotted with anti-β (mAb148) and anti-δ (mAb142) subunit antibodies (Santa Cruz Biotech.). Bound antibodies were detected using IRDye-conjugated anti-rat secondary antibody, imaged with an Odyssey Imaging System, and band intensities quantified using ImageStudio (Li-Cor). In experiments to assay AChR assembly, unassembled β subunit was isolated from cell extracts after isolation of assembled AChR, using mAb148 chemically cross-linked to protein G-agarose.

## Results

### Molecular Structure and Determinants of MX-Helix Retention Motifs

**FIGURE 1:**
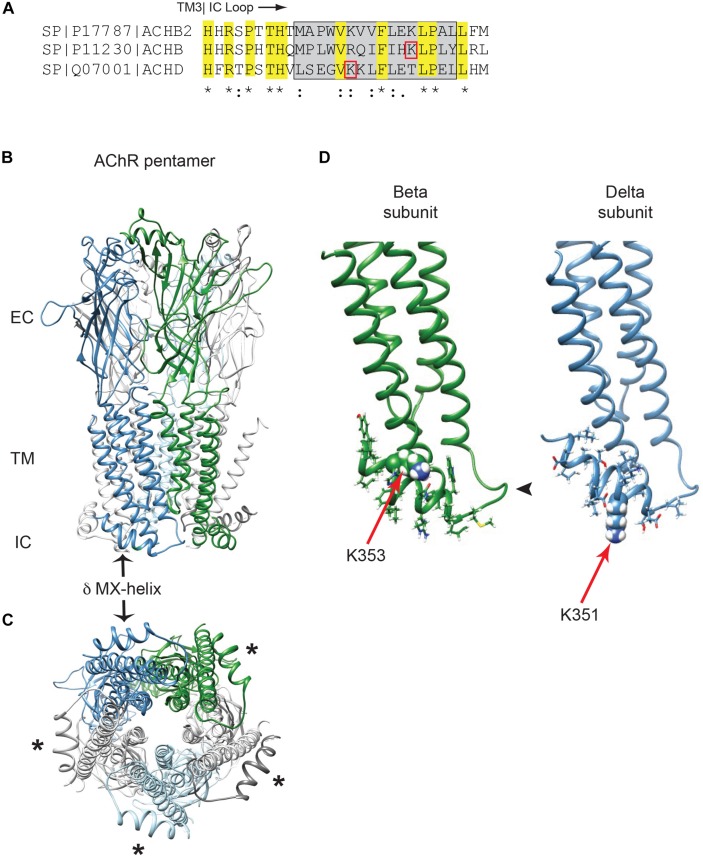
Golgi retention motifs are localized in the MX-helix. **(A)** Sequence alignment of the neuronal beta 2 and muscle beta and delta subunits, depicting the initial portion of the large cytoplasmic (TM3-TM4) loop. The MX-helix region is shown by the gray box, and the Golgi retention signals in β and δ are both centered on lysine residues (βK353 and δK351; denoted by red boxes). Sequence identity and conservation are represented by * and : symbols. **(B)** General architecture of the nicotinic acetylcholine receptor, based on the α4β2 crystal structure, showing the extracellular (EC), transmembrane (TM), and intracellular (IC) domains. Alpha subunits are in light gray, beta subunit in green, delta subunit in blue and epsilon subunit in light blue. The arrow marks the δ subunit MX-alpha helix, which is positioned laterally just below the membrane. Note that the rest of the intracellular domain is missing from the structure. **(C)** View perpendicular to the membrane looking from the intracellular side. Asterisks mark the MX-helix in each subunit. **(D)** The muscle beta and delta subunits were modeled using Rosetta structural modeling software. The initial portion of the TM3-TM4 cytoplasmic segment consists of a short loop following TM3 (arrowhead) followed by the laterally positioned MX-helix (residues shown with sidechains). The MX-helix contains the critical determinants for the Golgi retention signal, which are centered around K353 in the beta subunit and K351 in the delta subunit (red arrows mark key lysine residues, which are shown in space filling representation).

In previous work we identified Golgi retention motifs in the β and δ subunit proximal cytoplasmic loops (Rudell et al., 2014). Interestingly, this region corresponds to the MX-helix, which is a juxta-membrane amphipathic α-helix defined in recent crystal structures of cys-loop receptor family members (Hassaine et al., 2014; Morales-Perez et al., 2016). As this region is not resolved in the lower resolution structures of the muscle AChR (Unwin, 2005), we used Rosetta software to model the MX-helices using the neuronal α4β2 AChR crystal structure (Morales-Perez et al., 2016). As shown in Figures 1A–C, the structure and position of the MX-helix is similar in all subunits, although differences exist in the distribution of charged residues. In the case of the β and δ subunits, the Golgi retention motifs consist of a short loop following TM3 and the laterally positioned MX-helix (Figure 1D). The key sequence determinants for retention identified in our earlier study lie within the MX-helix and include an ordered sequence of hydrophobic and hydrophilic residues related to the partially amphipathic structure of the helix. Moreover, the critical lysine residues identified in the β and δ motifs (βK353 and δK351) are both surface-exposed in the model and potentially accessible, although the sidechains of βK353 are positioned close to the plasma membrane (Figure 1D). This MX helix structure provides a straightforward molecular basis for the functional effects described in our earlier mutational analysis (Rudell et al., 2014). For example, βK353L and δK351L mutations would locally alter the surface charge on the MX-helix, and other single amino-acid insertions or deletions that abolished retention (e.g., βQ348del, βL353ins, and δK354ins) would shift the rotational axis of the helix and also alter the relative position of the charged and hydrophobic residues. Together, this structural and functional analysis indicates that β and δ subunit trafficking motifs depend on the alpha-helical MX structure and its distribution of charged and hydrophobic residues.

**FIGURE 2:**
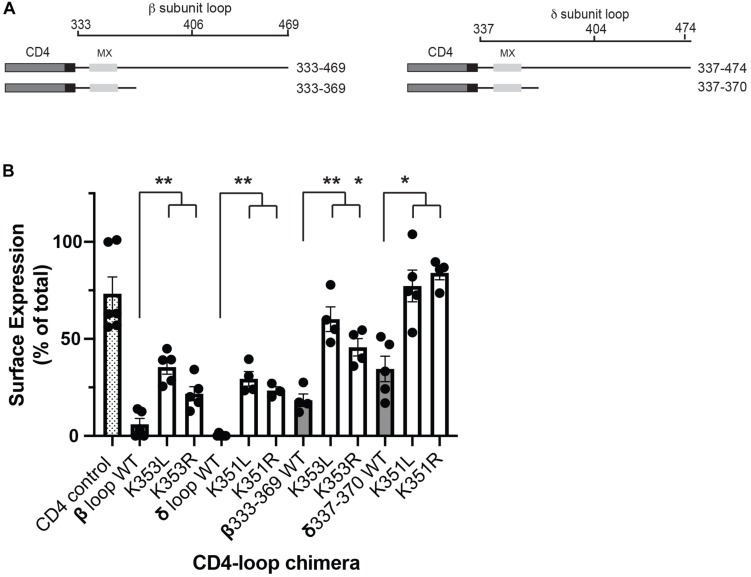
Molecular determinants for retention include specific lysine residues. **(A)** Schematic showing the CD4-subunit cytoplasmic loop chimeras used to characterize the retention signal. These consist of CD4 extracellular and transmembrane domains fused to either the full β or δ subunit intracellular loops, or to proximal loop fragments encompassing the MX-helix. **(B)** C2 mouse muscle cells were transfected in duplicate with each CD4-subunit loop chimera, and the percentage of each protein that was expressed on the cell surface was measured in on-cell Western blot assays. The percentage of CD4-β and δ full loops and CD4-β333-369 and δ337-370 on the cell surface (gray bars) was much lower than for CD4 control (hatched bar). Surface expression of the CD4-β and δ chimeras was increased significantly, however, by substitution of βK353 or δK351 for either leucine or arginine (**p < 0.01;*p < 0.05; one way ANOVA with Tukey’s multiple comparisons test; *n* = 4–6 experiments, error bars = SEM). The effect of K–L mutations tended to be slightly larger than K–R mutations, but this difference was not statistically significant.

Although all receptor subunits possess an MX-helix one feature of the β and δ subunits is the surface-exposed lysine residues (βK353 and δK351). To further probe their role in the retention signal, we compared the effects of mutating the lysine residues to either leucine or arginine. Substitution to leucine preserves the MX α-helical structure but replaces the positively charged lysine residue with a neutral amino acid, whereas substitution to arginine maintains the positively charged residue and thus the alignment of charged amino acids on the helix. For this, we expressed CD4-subunit loop chimeric proteins in C2 mouse muscle cells and used an on-cell western assay to quantify surface versus total expression for each chimera (Supplementary Figure 1). As shown in Figure 2A, the chimeras consist of the CD4 extracellular and transmembrane domains fused to the β or δ cytoplasmic loops, or to the minimal β or δ retention signals encompassing the MX-helix. As reported in our earlier study, a large fraction of CD4-β and δ loop chimeras were retained intracellularly, but mutating the key lysine residue in each motif to leucine (βK353L or δK351L) was sufficient to increase their surface expression several-fold (Rudell et al., 2014). Surprisingly, we found that lysine to arginine mutations also significantly increased their surface expression (Figure 2B), with surface levels approaching those of CD4-loop chimeras with K-L mutations. Indeed, β K353L and K353R mutations increased surface levels of CD4-β loop by 5.9 and 3.6-fold, and CD4-β333-369 by 3.2 and 2.5-fold, respectively. Similarly, δ K351L and K351R mutations increased surface levels of CD4-δ loop by 98 and 77-fold, and CD4-δ337-370 by 2.2 and 2.9-fold. These experiments indicate that the β and δ retention signals depend not only on the amphipathic nature of the MX-helix, but also on specific lysine residues (βK353 and δK351).

### Role of the MX-Helix Retention Motifs in Quality Control

**FIGURE 3:**
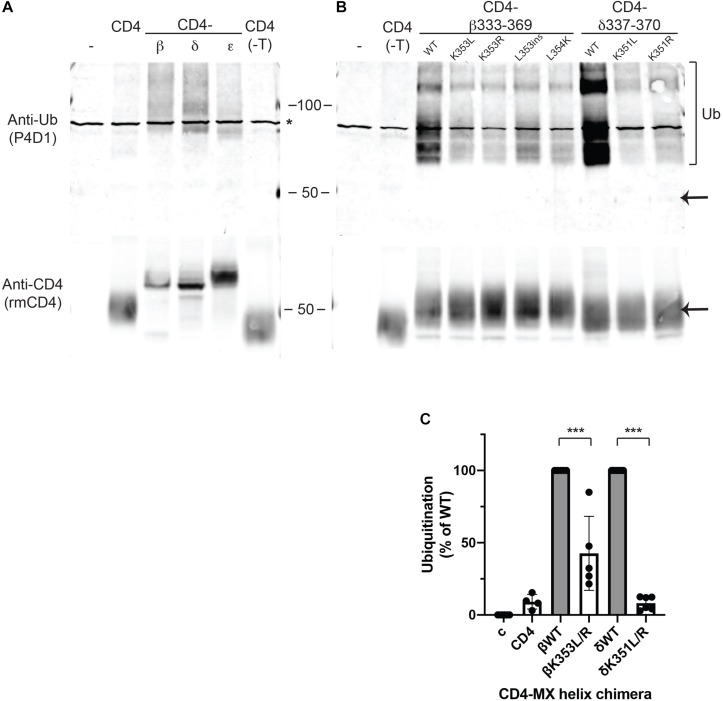
Retention is associated with ubiquitination of MX-helix lysine residues. **(A,B)** CD4 subunit loop chimeras expressed in HEK cells were immunoprecipitated from cell extracts using anti-CD4 antibody cross-liked to agarose beads. The isolates were immunoblotted with monoclonal anti-ubiquitin antibody (P4D1), and then re-probed with polyclonal anti-CD4 antibody. We detected ubiquitination of both CD4-β and δ full loops **(A)** and CD4-β333-369 and δ337-370 MX-helix chimeras **(B)**. The ubiquitination was evident as a characteristic ladder of high molecular weight forms (bracket) beginning around 9 kD above the major band detected with anti-CD4 antibody (arrow). In contrast, little or no ubiquitination was evident for CD4 (control), CD4 with a deleted intracellular tail (CD4-T), or CD4-ε loop. Note that the asterisk marks a non-specific band present in non-transfected samples. **(B)** High levels of ubiquitination of CD4-β333-369 and δ337-370 were greatly reduced by mutation of βK353 or δK351 to either leucine (L) or arginine (R). Mutations adjacent to βK353 also reduced ubiquitination (βL353ins and βL354K). **(C)** Quantification of the immunoblots shows that ubiquitination of CD4-β333-369 was reduced 57% by K353L/R mutations, and ubiquitination of CD4-δ337-370 was reduced 92% by K351L/R mutations (***p < 0.001, one way ANOVA with Tukey’s multiple comparison’s test, *n* = 4–6 independent transfection experiments).

Lysine residues are notable in that they are targets for several types of post-translational modification. The most common is ubiquitination, which plays an important role in the regulation of trafficking, degradation and quality control of many membrane proteins (Mukhopadhyay and Riezman, 2007; MacGurn et al., 2012; Foot et al., 2017). Consequently, we tested whether MX-helix lysine residues are ubiquitinated and if ubiquitination contributes to retention. For this, we expressed CD4-subunit loops in HEK cells as the low expression levels of some chimeras in muscle cells was prohibitive for biochemical experiments. The CD4-subunit loop chimeras were then immunoprecipitated from cell extracts using anti-CD4 antibody and immunoblotted with anti-ubiquitin antibody. Notably, we detected significant ubiquitination of CD4-β and δ loops but low or undetectable ubiquitination of CD4 or CD4-ε loops (Figure 3A). The ubiquitination of CD4-β and δ loops was evident as a higher molecular weight ladder beginning around 9 kD above the major CD4-β and δ bands detected with anti-CD4 antibody, consistent with the addition of one or more ubiquitin molecules. Thus, ubiquitination occurs on CD4-β and δ loops which are retained intracellularly, but not on CD4 and CD4-ε loop, which are trafficked efficiently to the cell surface.

To then define the determinants for ubiquitination, we expressed CD4-β and δ proximal loop fragments with mutations of βK353 and δK351 or of neighboring residues. We detected robust ubiquitination of CD4-β333-369, which was significantly reduced by mutation of K353 to either L or R (Figure 3B). Similarly, ubiquitination of CD4-δ337-370 was largely abolished by mutation of K351 to L or R and comparable to CD4 control (Figures 3B,C). In addition, we tested whether other mutations previously found to impair retention of CD4-β333-369 also reduced ubiquitination. Indeed, insertion of a leucine in the MX helix (L353ins) or substitution of L354 for lysine both greatly decreased ubiquitination, despite the presence of K353 or even an additional lysine. Finally, by immunoblotting with anti-CD4 antibody we confirmed that the differing levels of ubiquitination were unrelated to total expression of CD4-β333-369 and δ337-370, and also that only a small proportion of each occurred in the higher molecular weight, ubiquitinated form. Together, these findings suggest that ubiquitination occurs largely on βK353 and δK351 and is dependent on a specific recognition sequence that encompasses these lysines in the MX-helix. Ubiquitination correlates closely with intracellular retention of the CD4-β and δ loop chimeras, and likely regulates either their retention in the Golgi apparatus and/or their subsequent degradation. Thus, ubiquitination of the MX-helix motifs contributes to Golgi-based quality control of receptor trafficking.

**FIGURE 4:**
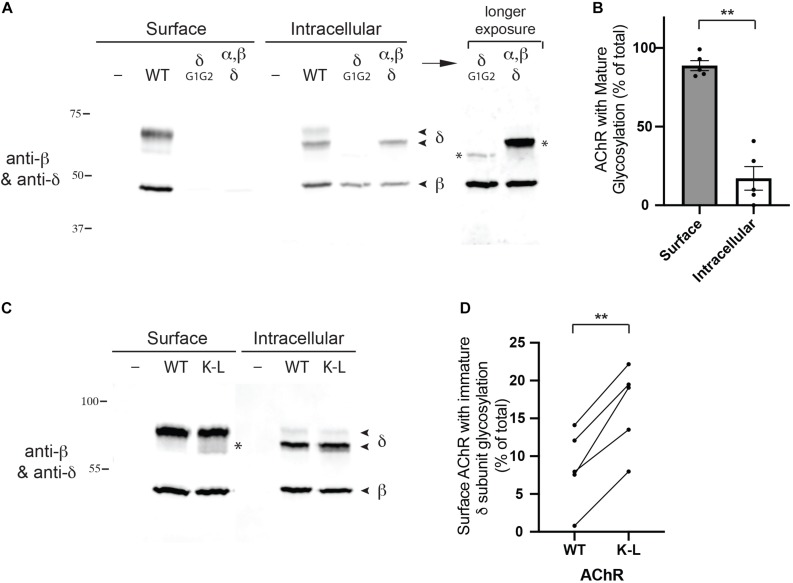
Mutation of the Golgi retention motif increases surface AChR with immature glycosylation. **(A)** HEK cells were transfected with wild type AChR subunits (α, β, ε, and an epitope-tagged δ subunit), or with AChR containing δ subunit with mutated N-linked glycosylation sites. We then sequentially isolated surface and intracellular AChR and immunoblotted the isolates for the β and δ subunits. We found that surface AChR contained a higher molecular weight form of δ subunit compared to intracellular AChR (∼68 cf. 62 kD), which has been shown to be due to modification of N-linked oligosaccharides during transit through the Golgi complex. Consistent with this, AChR containing δ with mutated glycosylation sites (δG1G2), or only α, β and δ subunits were largely retained in the intracellular receptor pool and contained only lower molecular weight forms of δ (marked by asterisks in longer exposure image). **(B)** Quantification shows that surface AChR contains largely high molecular weight δ subunit with mature glycosylation (89% of total). In contrast, intracellular AChR contains only 17% high molecular weight δ subunit, with the vast majority (83%) being low molecular weight δ subunit with immature glycosylation (**p < 0.005; paired t test; *n* = 5 experiments; error bars = SEM). **(C)** HEK cells were transfected with wild type AChR or AChR containing combined βK353L and δK351L mutations (K–L), and surface and intracellular AChR were sequentially isolated and immunoblotted with anti-β and δ subunit antibodies. Compared to wild type AChR, mutant AChR contained significantly more low molecular weight delta subunit with immature glycosylation (asterisk). **(D)** Quantification showing the percentage of WT and K–L surface AChR with immature δ subunit glycosylation, with each pair of data points representing a separate transfection experiment. Combined βK353L/δK351L mutations increased the amount of surface AChR with immature glycosylation by twofold, from 8 to 16% (**p < 0.005; paired *t*-test; *n* = 5 experiments).

Next, we tested whether the Golgi retention motifs also regulate trafficking and quality control of fully assembled AChR. For this, we focused on glycosylation of the AChR, which includes a key maturational step that occurs in the Golgi apparatus. Previous studies have shown all receptor subunits contain conserved N-linked glycosylation sites mostly with simple oligosaccharides added during receptor biosynthesis in the ER (Gehle et al., 1997). In the case of the gamma and delta subunits, however, the simple oligosaccharides at one of the glycosylation sites are modified to complex forms in the Golgi apparatus (Gu and Hall, 1988; Ramanathan and Hall, 1999), prior to receptor trafficking to the plasma membrane. First, to confirm the earlier studies, we sequentially isolated surface and intracellular AChR from transfected HEK cells (see section “Methods”), and then immunoblotted for the β and δ subunits (Figure 4A). To unambiguously identify the δ subunit we utilized an antibody to a C-terminal epitope tag, as available antibodies recognize both the γ and δ subunits. We found that the wild type (WT) δ subunit in surface AChR runs at significantly higher molecular weight than δ in intracellular AChR (∼68 cf. 62 kD), confirming that its N-linked oligosaccharides are modified prior to trafficking to the plasma membrane. Indeed, quantification of these experiments shows that ∼90% of the delta subunit in surface AChR has mature glycosylation, compared to only 17% in intracellular receptor (Figure 4B). To confirm that the shift in molecular weight was due to changes in glycosylation we expressed AChR containing delta subunit with mutations of the glycosylation sites (δG1G2). As expected, deltaG1G2 was evident as a single band of lower molecular weight and present only in intracellular receptor (Figure 4A). Similarly, expression of an incomplete set of receptor subunits (α, β, and δ subunits) resulted in delta subunit with largely immature glycosylation and mostly in intracellular receptor. These findings confirm earlier studies and show that N-linked glycosylation of the delta subunit is required for efficient subunit assembly and surface expression of AChR.

We have shown previously that mutation of the retention motifs increases surface AChR by promoting Golgi to surface trafficking (Rudell et al., 2014). Consequently, we tested whether the β/δ retention motifs contribute to delta subunit glycosylation in the Golgi and if their mutation perturbs the selective expression of mature AChR on the cell surface. To do this, we expressed AChR in wild type form or with combined βK353L and δK351L mutations, and sequentially isolated surface and intracellular receptor and then immunoblotted for the β and δ subunits. We found that surface wild type and mutant AChR contained predominantly the high molecular form of the delta subunit with mature glycosylation. However, a significantly higher percentage of low molecular weight delta subunit was detected in βK353L/δK351L – AChR compared to wild type AChR (16% cf. 8%, Figures 4C,D). Thus, mutation of the retention motifs increases surface AChR with immature glycosylation by twofold, indicating that the motifs contribute to Golgi trafficking and quality control of assembled receptor.

### Role of MX-Helix in AChR Assembly and Expression

**FIGURE 5:**
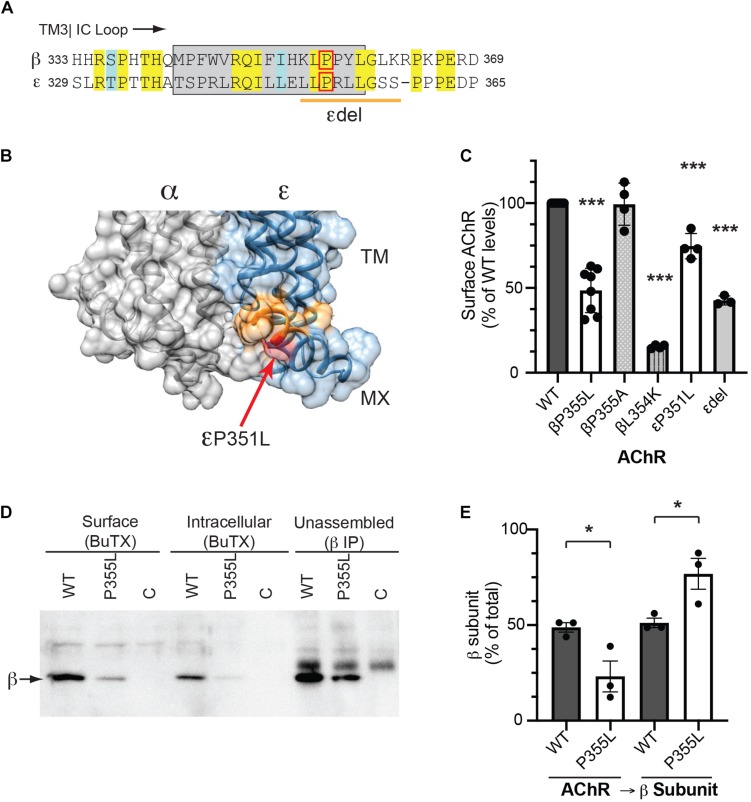
CMS-linked mutations in the MX-helix impair AChR assembly and expression. **(A)** Sequence alignment showing the initial portion of the beta and epsilon cytoplasmic (TM3-TM4) loops, with the MX-helix shown by the gray box. The two CMS-linked mutations in the epsilon subunit are denoted by the red box (εP351L) and orange line (εdel349-357), respectively. The analogous proline in the β subunit MX-helix (βP355L) is indicated by the red box. **(B)** Molecular structure showing the cytoplasmic interface between the alpha and epsilon subunits. The end of the ε MX helix contacts the short loop connecting TM3 to the MX-helix of the α subunit. Residues deleted in the εdel mutation are shown in orange and the εP351L mutation is marked by the red arrow. **(C)** HEK cells were transfected with AChR in wild type form or with the indicated mutations in the MX-helix, and surface levels of AChR were measured by ^125^I-alpha-BuTx binding. Compared to WT AChR, the CMS-linked mutations in epsilon (εP351L and εdel349-357) decreased surface levels of AChR by 25 and 57%, respectively. The corresponding βP355L mutation decreased surface AChR by 51% but βP355A mutation had no discernable effect. Beta L354K mutation, which is predicted to misposition the MX helix, decreased surface AChR by 85% (***p < 0.0001 as compared to WT; one way ANOVA with Dunnett’s multiple comparison’s test; *n* = 3–8 experiments). **(D)** HEK cells were transfected with wild type or βP355L-AChR. We then sequentially isolated surface AChR, intracellular AChR, and unassembled beta subunit, and immunoblotted with anti-beta subunit antibody to compare the levels in each pool. Significant amounts of wild type β subunit were detected in the pools of surface and intracellular AChR, as well as in the pool of unassembled subunit. In contrast, βP355L subunit was mostly detected in the unassembled subunit pool, indicating that it assembled less efficiently into AChR. **(E)** Quantification shows that a smaller percentage of P355L β subunit (23%) assembled into AChR compared to wild type β subunit (49%) (*p < 0.05, one way ANOVA with Sidak’s multiple comparison’s test, *n* = 3 experiments).

The MX-helix is present in all receptor subunits and could regulate additional aspects of AChR assembly or trafficking that are critical for its expression and function at the NMJ. Consistent with this, two previously identified human mutations linked to congenital myasthenic syndrome (CMS) localize to the epsilon subunit MX-helix. The first is a homozygous missense mutation (CHRNE P351L; Croxen et al., 2001), which reduces assembly and surface expression of AChR in heterologous cells, and the second is a homozygous 27 base pair deletion (CHRNE 1046_1072del27 eliminating amino acids 349-357), whose functional effects are uncharacterized (Maselli et al., 2010). Interestingly, these mutations occur near the end of the MX helix (Figure 5A) and the structural model shows that these residues contribute to the cytoplasmic interface between the α and ε subunits (Figure 5B). Indeed, in the molecular structures of both the α4β2 and 5HT_3_ receptors, the end of each subunit MX-helix contacts the short loop following TM3 in the adjacent subunit (Hassaine et al., 2014; Morales-Perez et al., 2016). These findings suggest that the MX-helix could play a role in subunit assembly and AChR expression. To test this, we compared the effects of the CMS-linked mutations in both the epsilon and beta subunits. For this, wild type and mutant AChR subunits (α, β, ε, and δ) were expressed in heterologous HEK cells, and levels of surface receptor were measured by binding of I^125^-labeled alpha-bungarotoxin. Compared to wild type AChR, we found that surface receptor levels were decreased 25% for εP351L-AChR and 55% for εdel349-357-AChR (Figure 5C). An analogous mutation in the β subunit (βP355L) decreased receptor levels by 50%, but a βP355A mutation had no discernable effect. In addition, we found that a targeted mutation of the preceding residue (βL354K) designed to misposition the MX-helix reduced surface receptor levels by 85%. In the case of CHRNE P351L, the decrease in surface receptor results primarily from impaired assembly of the mutant subunit (Croxen et al., 2001). To test whether this is also the case for βP355L we sequentially isolated assembled AChR and then unassembled beta subunit and immunoblotted to compare the amount of beta subunit in each pool (Figure 5D). Notably, βP355L subunit assembled into AChR much less efficiently than wild type β subunit (Figure 5E). Together, these findings show that CMS mutations in the epsilon subunit and analogous mutations in the beta subunit both reduce surface expression of the AChR, in large part by impairing subunit assembly. Thus, the MX-helix also contains shared determinants that contribute to subunit assembly and AChR expression.

## Discussion

AChR function at the NMJ depends on its appropriate assembly, processing and trafficking to the cell surface, with each step in receptor biogenesis being regulated by specific molecular determinants in the receptor subunits. Here, we identify important regulatory signals localized in the MX-helix, which is a juxta-membrane α-helix present in the proximal, major cytoplasmic loop of all subunits. First, specific sequence determinants in the β and δ subunit MX-helices mediate Golgi retention and contribute to quality control of AChR expression by (i) retention of unassembled subunit loops, leading to their ubiquitination and degradation, and (ii) retention of assembled AChR, helping ensure appropriate glycosylation occurs in the Golgi prior to their surface trafficking. Second, shared determinants in a distinct region of the subunit MX-helices contribute to subunit assembly, as CMS and targeted mutations near the end of the MX-helix impair receptor assembly and surface expression.

### Molecular Signals in MX Helix

The Golgi retention motifs that we previously identified in the β and δ subunits reside in the MX-helix, which was defined in crystal structures of both the α4β2 neuronal AChR and the 5HT_3_ receptor (Hassaine et al., 2014; Morales-Perez et al., 2016). This juxta-membrane α-helix is present in all subunits and is positioned laterally in the proximal TM3-TM4 cytoplasmic loop, placing it in close proximity to the inner leaflet of the plasma membrane, and ending near the interface with the adjacent receptor subunit. Using the neuronal α4β2 structure we generated a molecular model of the muscle β and δ subunit motifs, whose structure readily accounts for the sequence requirements for Golgi retention identified in our earlier mutational analysis (Rudell et al., 2014). First, the requirement for an ordered array of charged and hydrophobic residues corresponds directly to the amphipathic nature of the helix, and explains why single amino-acid insertions, deletions, or substitutions of hydrophilic and hydrophobic residues all reduced intracellular retention. Second, the critical lysine residues in β and δ (βK353 and δK351) are both surface exposed with outward-facing sidechains, whereas lysine residues are absent at the equivalent positions in the other subunits (α, γ, and ε). The importance of βK353 and δK351 is further underscored by our finding that even substitution with another positively charged residue (arginine) significantly reduced Golgi retention. Together with our earlier findings, this suggests that the Golgi retention signal consists of a short, solvent-exposed face of the MX-helix encompassing the key lysine residue.

The MX-helix structure is conserved in all subunits of both the α4β2 AChR and 5HT_3_ receptors, however, consistent with additional functions in AChR assembly and function (Wu et al., 2015). Indeed, we found that CMS-linked mutations in epsilon (εP351L and εdel349-357) that reduce AChR assembly and levels are located at the end of the MX-helix, and our structural model shows that these residues contact the initial portion of the alpha subunit TM3-TM4 loop. An analogous P355L mutation in the β subunit MX-helix also impaired subunit receptor assembly and expression (Figure 5), whereas a P355A mutation had no detectable effect on either. The reason for this remains unclear, but potentially the longer sidechains of leucine could disrupt either the folding and/or inter-subunit interactions of the beta subunit. Notably, this proline residue is part of a highly conserved (L/I/V)P sequence pair, which is present in almost all muscle and neuronal AChR subunits and consequently could contribute generally to the cytoplasmic interface between subunits and their assembly. Moreover, we have shown previously that the LP residues are not required determinants for the β/δ Golgi retention signals (Rudell et al., 2014). Thus, these findings define additional molecular determinants in the MX-helix important for receptor assembly/expression, which map to the end of the MX-helix and are distinct from the Golgi retention signals.

### Function and Mechanism of MX-Helix Signals

The retention signals specific to the β/δ subunit MX-helix regulate Golgi trafficking and we propose that they contribute to quality control of AChR expression in two ways. First, our findings suggest that the retention motifs help prevent surface expression of unassembled or incorrectly assembled receptor subunits that escape the ER quality control mechanisms. The presence of an additional quality control checkpoint at the Golgi complex has been documented in many systems (Arvan et al., 2002; MacGurn et al., 2012), and some studies have suggested such a checkpoint exists in AChR trafficking to the cell surface (Keller et al., 2001; Zhao et al., 2009; Rezvani et al., 2010; Crespi et al., 2018). The Golgi-resident protein that binds the β/δ motifs remains unknown, but is likely distinct from known proteins such as unc-50 or VILIP which both promote Golgi to surface trafficking of the receptor (Eimer et al., 2007; Zhao et al., 2009). It is also unlikely to be the sorting receptor Rer1p, which recognizes and retains unassembled α subunits (Valkova et al., 2011), rather than a motif specific to the β and δ subunits. The mechanism by which Golgi protein binding to the β/δ motifs discriminates between unassembled subunits and assembled receptor, and thereby provides quality control also remains unclear. One possibility is that the motifs are exposed in unassembled or incorrectly assembled subunits, leading to binding and retention, but are partially masked or regulated in correctly assembled receptor, permitting forward trafficking to the plasma membrane. Masking could occur through inter-subunit contacts or conformational changes that reduce the accessibility of the motif. For example, our molecular model suggests that appropriate assembly of the β subunit into pentameric receptor would position βK353 close to the inner leaflet of the plasma membrane (see Figure 1), which could potentially mask the β MX-helix retention motif.

Additional support for a quality control function comes from our finding that Golgi retention is associated with ubiquitination of the β/δ MX-helix motifs. Indeed, we observed robust and selective ubiquitination of CD4-β and δ MX-helices, which was largely abolished by mutation of the key lysine residues in each (βK353 and δK351). Thus, we propose that these lysines are the predominant sites of ubiquitination, and consistent with this, βK353 has been found to be ubiquitinated in proteomic studies (Hornbeck et al., 2015). As point mutations of neighboring residues also impaired ubiquitination, ubiquitination of βK353 or δK351 likely involves recognition of a specific amino-acid sequence by an E3 ubiquitin ligase. Potential candidates are the E3 ligases PDZRN3, MuRF1, NEDD4, and TRIM63, which have all been implicated in protein turnover at the NMJ (Lu et al., 2007; Liu et al., 2009; Rudolf et al., 2013; Khan et al., 2014). Golgi-based ubiquitination has previously been shown to sort mis-folded proteins into degradation pathways (MacGurn et al., 2012), and it seems likely that ubiquitination of the MX-helix motifs acts in a similar manner and targets unassembled or incorrectly assembled subunits for degradation via either the lysosomal or proteosomal pathways (Foot et al., 2017). One key question, however, is whether ubiquitination is the signal for retention, or a consequence of retention that marks the proteins for degradation. We tend to favor the latter idea for several reasons: (i) in immunostaining experiments we detected significant accumulation of CD4-β/δ chimeras in the Golgi but no corresponding accumulation of ubiquitinated protein; (ii) in immunoblotting experiments, we found that only a small minority of CD4-β/δ chimeras is ubiquitinated (i.e., evident as higher molecular weight bands); and (iii) in immunostaining experiments we still observed some accumulation of CD4-βK353R MX-helix in the Golgi, despite little or no ubiquitination of this chimera. Thus, we propose that unassembled or incorrectly assembled subunits that escape the ER are retained in the Golgi through recognition of the β/δ motifs by a Golgi-resident protein and that this leads to their ubiquitination and degradation.

Second, our findings demonstrate that the β/δ motifs also contribute to quality control of assembled AChR by regulating its glycosylation in the Golgi complex. After receptor subunits are synthesized and assembled in the ER, the AChR is trafficked to the Golgi complex for further processing, which includes the modification of N-linked oligosaccharides on the γ and δ subunits to more complex forms. This process is normally highly efficient, as almost all surface receptor contains delta subunit with mature glycosylation. We found, however, that mutation of the β and δ subunit retention motifs (with combined βK353L/δK351L mutations) resulted in a significant 2-fold increase in the proportion of surface receptor with immature glycosylation. Thus, we propose that the β/δ motifs help retain the receptor in the Golgi to allow for modification of the oligosaccharides to complex forms. This is consistent with our earlier finding that mutation of the β/δ motifs increased surface levels of AChR (Rudell et al., 2014). Moreover, it implies that at least one of the retention motifs remains accessible in assembled AChR, although it may be regulated to permit Golgi to surface trafficking. These findings define a second form of quality control overseeing maturational steps in assembled AChR, and may have important functional consequences as N-linked glycosylation significantly affects AChR channel function and surface expression (Gehle et al., 1997; Ramanathan and Hall, 1999; Nishizaki, 2003; Dellisanti et al., 2007). Interestingly, a different form of Golgi-based quality control was recently demonstrated for the neuronal AChR, where receptors with α3β4α5 stoichiometry were found to be selectively recognized in the Golgi and recycled, whilst (α3)2(β4)3 receptors were trafficked to the cell surface (Crespi et al., 2018). Together with our current findings, this demonstrates that trafficking of assembled AChR is regulated at the Golgi complex and that this checkpoint governs additional aspects of AChR biogenesis and maturation.

Finally, in addition to regulating Golgi trafficking, we show that the MX-helix plays a role in receptor assembly. Two CMS-linked mutations in the epsilon subunit MX-helix (εP351L and εdel349-357) significantly reduced surface expression of the receptor, which is likely due to impaired α/ε heterodimer formation (Croxen et al., 2001). Similarly, we found that the corresponding mutation introduced into the beta subunit MX-helix (βP355L) also reduced AChR levels by inhibiting β subunit assembly, as did a βL354K mutation predicted to misposition the MX-helix. Notably, these mutations lie at the end of the MX-helix, which in the α4β2 crystal structure contacts the short loop following TM3 in the adjacent subunit. In addition, the end of the MX-helix may facilitate the correct folding of the following residues, which were not resolved in the crystal structure, but could also contribute to the interface between adjacent subunits. Consequently, these mutations likely directly disrupt subunit recognition and assembly, although we cannot discount the possibility that they perturb subunit folding and indirectly inhibit subunit assembly. These findings suggest, therefore, that shared determinants in the subunit MX-helices contribute to subunit assembly and are important for AChR surface expression.

**FIGURE 6:**
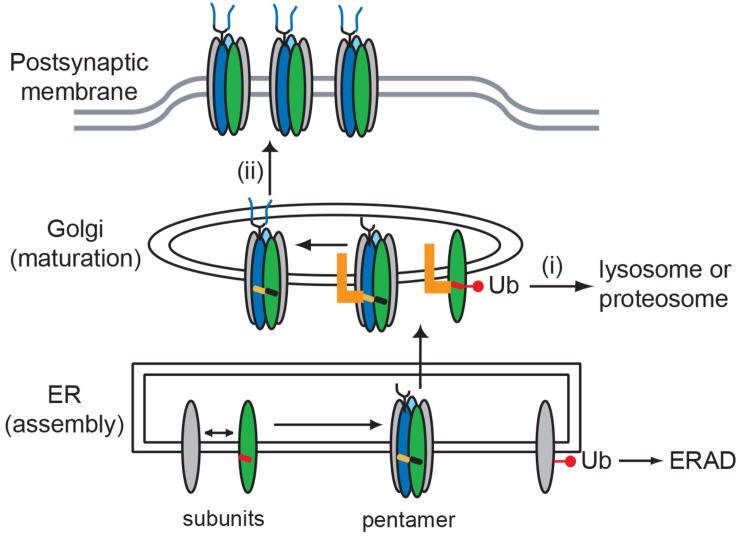
Model for role of MX-helix in AChR assembly and trafficking. Our findings identify two molecular signals in the AChR subunit MX-helix that regulate receptor assembly and trafficking. First, shared determinants near the end of the MX-helix contribute to receptor assembly in the ER. Second, specific determinants in the β (green) and δ (blue) subunit MX-helix regulate Golgi trafficking and quality control by: (i) retention of incorrectly assembled subunits that escape endoplasmic reticulum associated protein degradation (ERAD), which leads to their ubiquitination and degradation; and (ii) transient retention of assembled AChR, which facilitates the modification of N-linked glycosylation (oligosaccharides depicted in blue). The molecular basis for the two forms of quality control could stem from the motifs being exposed in incorrectly assembled receptor (red MX-helix), but being masked or regulated in assembled receptor (black and orange MX-helices). The identity of the Golgi-resident protein that binds the motifs (orange L) remains unknown.

In summary, we define roles for the MX-helix in both AChR subunit assembly and Golgi trafficking and quality control. These functions are potentially linked as shown in our working model in Figure 6. We propose that incorrect or incomplete assembly of receptor subunits mispositions the MX-helix and exposes the retention signals, leading to their ubiquitination and sorting into degradative pathways. In contrast, correct assembly of receptor subunits positions the MX-helix so that the retention motifs are partially masked or regulated, and thereby allows forward trafficking of the AChR. Together with earlier ER-based quality control mechanisms, this would ensure that only correctly assembled – fully functional – AChR is trafficked to the muscle plasma membrane and localized at the NMJ.

## Data Availability Statement

All datasets generated for this study are included in the article/Supplementary Material.

## Author Contributions

VY-Y performed the Rosetta structural modeling. JR and LB performed the experiments, analyzed the data, and edited the manuscript. MF performed the experiments, analyzed the data, and wrote the manuscript.

## Conflict of Interest

The authors declare that the research was conducted in the absence of any commercial or financial relationships that could be construed as a potential conflict of interest.
