# Supplementary figures and images for: The MX-Helix of Muscle nAChR Subunits Regulates Receptor Assembly and Surface Trafficking
Source: Front Mol Neurosci. 2020 Mar 24;13:48. doi: 10.3389/fnmol.2020.00048 (PMC7105636; doi:10.3389/fnmol.2020.00048)

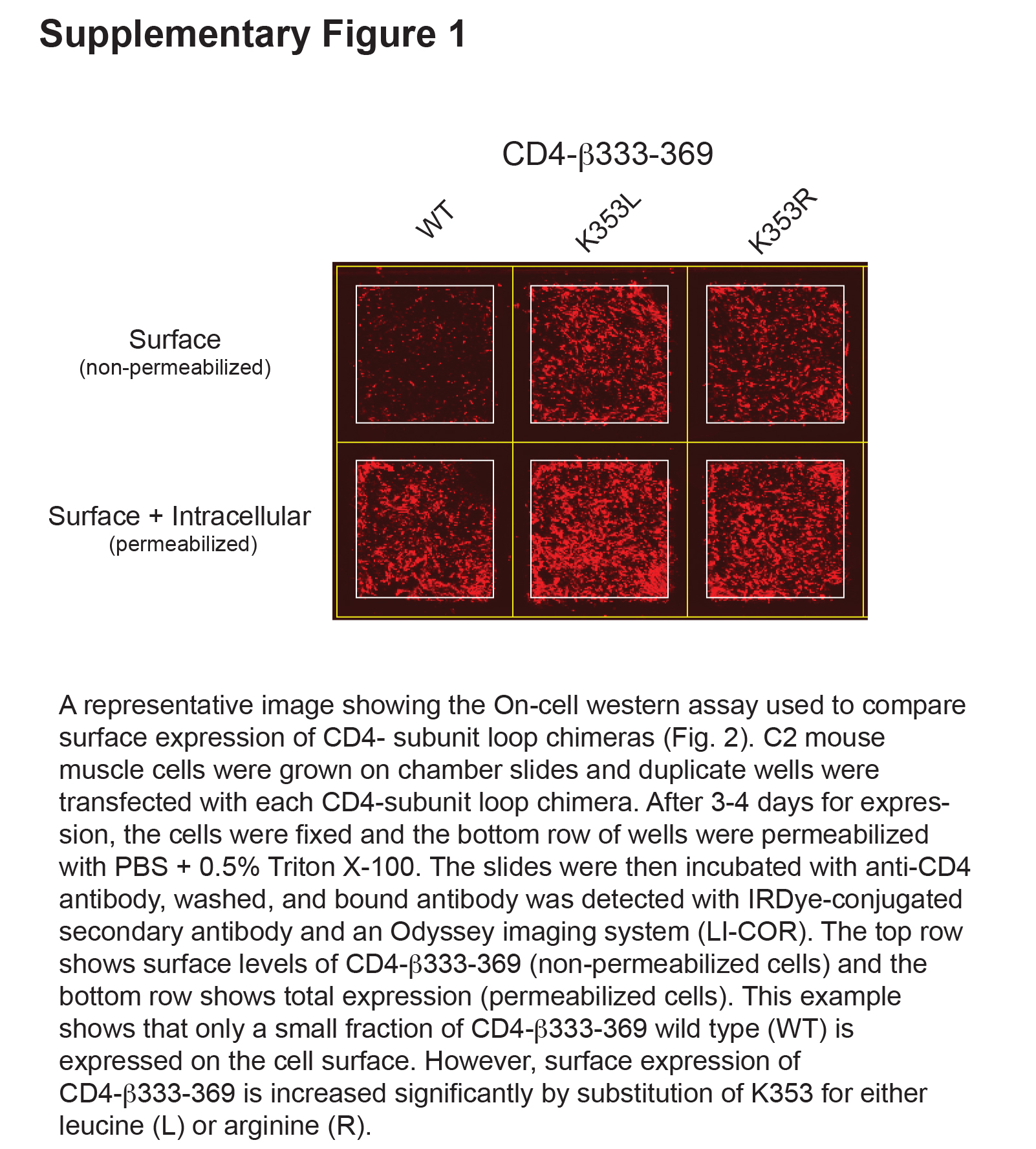

Supplement: Supplementary file 1 [file Image_1.JPEG]
